# Supplementary material for: Hypertensive Disorders of Pregnancy (HDP) and the Risk of Common Cancers in Women: Evidence from the European Prospective Investigation into Cancer (EPIC)-Norfolk Prospective Population-Based Study
Source: Cancers (Basel). 2020 Oct 23;12(11):3100. doi: 10.3390/cancers12113100 (PMC7690818; doi:10.3390/cancers12113100)
Supplement: Supplementary file 1 [file cancers-12-03100-s001.pdf]

cancers

Supplementary Materials

Hypertensive Disorders of Pregnancy (HDP) and the Risk of Common Cancers in Women: Evidence from the European Prospective Investigation into Cancer (EPIC)-Norfolk Prospective Population-Based Study

Zahra Pasdar, David T Gamble, Phyo K Myint, Robert N Luben, Nicholas J Wareham, Kay-Tee Khaw and Sohinee Bhattacharya

Table S1. Baseline characteristics of 13,562 women of the EPIC-Norfolk according to incident breast cancer status and corresponding unadjusted and adjusted odds ratios with 95% CIs

	Incident breast cancer		Unadjusted OR (95% CI)	P-value	Adjusted OR* (95% CI)	P-value
	No [n=12860]	Yes [n=702]				
**Median age years (IQR) [n=13562]**	**58.1 (16.2)**	**57.1 (14.1)**	**0.99 (0.98-1.00)**	0.018	**0.99 (0.98-1.00)**	0.023
**Education (%) [n=13562]**						
**No qualification**	5807 (45.2)	290 (41.3)	Ref		Ref	
**0 Level**	1512 (11.8)	78 (11.1)	1.03 (0.80-1.34)	0.137	0.93 (0.71-1.21)	0.662
**A Level**	4334 (33.7)	262 (37.3)	**1.21 (1.02-1.44)**		1.07 (0.88-1.29)	
**Degree**	1207 (9.4)	72 (10.3)	1.19 (0.92-1.56)		0.95 (0.70-1.28)	
**Occupational Social Class (%) [n=13190]**						
**Professional**	770 (6.0)	50 (7.1)	Ref		Ref	
**Manager**	4153 (32.3)	232 (33.0)	0.86 (0.63-1.18)		0.89 (0.65-1.23)	
**Skilled Non-manual**	2395 (18.6)	130 (18.5)	0.84 (0.60-1.17)	0.675	0.92 (0.65-1.30)	0.951
**Skilled Manual**	2796 (21.7)	158 (22.5)	0.87 (0.63-1.21)		0.98 (0.69-1.38)	
**Semi-Skilled**	1816 (14.1)	89 (12.7)	0.76 (0.53-1.08)		0.86 (0.59-1.26)	
**Non-Skilled**	573 (4.5)	28 (4.0)	0.75 (0.47-1.21)		0.90 (0.55-1.48)	
**Missing**	357 (2.8)	15 (2.1)				
**Smoking History (%) [n=13441]**						
**Never**	7094 (55.2)	384 (54.7)	Ref		Ref	
**Ex-smoker**	4092 (31.8)	80 (11.4)	0.95 (0.74-1.21)	0.886	1.03 (0.86-1.22)	0.965
**Currently smoking**	1559 (12.1)	232 (33.0.)	1.05 (0.89-1.24)		0.96 (0.74-1.23)	
**Missing**	115 (0.9)	6 (0.9)				

**Alcohol (g) (%) [n=11035]**						
**0**	2584 (20.1)	127 (18.1)	Ref		Ref	
**0.1-4.9**	4111 (32.0)	211 (30.1)	1.04 (0.83-1.31)		1.00 (0.79-1.25)	
**5-14.9**	2907 (22.6)	167 (23.8)	1.17 (0.92-1.48)	0.009	1.10 (0.86-1.41)	0.020
**15-29.9**	629 (4.9)	52 (7.4)	**1.68 (1.20-2.35)**		**1.61 (1.14-2.28)**	
**≥30**	227 (1.8)	20 (2.8)	**1.79 (1.10-2.93)**		**1.72 (1.04-2.84)**	
**Missing**	2402 (18.7)	125 (17.8)				
**Physical activity (%) [n=13562]**						
**Active**	4786 (37.2)	269 (38.3)	Ref	0.556	Ref	0.978
**Inactive**	8074 (62.8)	433 (61.7)	0.95 (0.82-1.12)		1.00 (0.85-1.18)	
**BMI (kg/m²) (%) [n=11414]**						
**Normal**	4727 (36.8)	222 (31.6)	Ref		Ref	
**Underweight**	71 (0.6)	1 (0.1)	0.30 (0.04-2.17)		0.31 (0.04-2.23)	
**Pre-obesity**	4183 (32.5)	272 (38.7)	**1.39 (1.15-1.66)**	0.005	**1.47 (1.22-1.76)**	0.001
**Obesity Class I**	1402 (10.9)	67 (9.5)	1.02 (0.77-1.35)		1.11 (0.83-1.47)	
**Obesity Class II +**	439 (3.4)	30 (4.3)	1.46 (0.98-2.16)		1.59 (1.06-2.38)	
**Missing**	2038 (15.8)	110 (15.7)				
**Family history of cancer (%) [n=5295/13561]**	4989 (38.8))	306 (43.6)	**1.22 (1.05-1.42)**	0.041	**1.21 (1.04-1.41)**	0.055
**Missing**	1 (0.0)	0 (0.0)				
**Prevalent MI (%) [n=200/13557]**	191 (1.5)	9 (1.3)	0.86 (0.44-1.69)	0.361	1.02 (0.52-2.02)	0.294
**Missing**	4 (0.0)	1 (0.1)				

**Prevalent stroke (%) [n=144/13559]**	140 (1.1)	4 (0.6)	0.52 (0.19-1.41)	0.439	0.59 (0.22-1.61)	0.592
**Missing**	3 (0.0)	0 (0.0)				
**Prevalent Diabetes (%) [n=226/13557]**	218 (1.7)	8 (1.1)	0.67 (0.33-1.36)	0.538	0.72 (0.35-1.47)	0.664
**Missing**	5 (0.0)	0 (0.0)				
**Hypertension during pregnancy (%) [n =2919/12008]**	86203 (67.0)	469 (66.8)	1.09 (0.91-1.31)	0.237	1.06 (0.88-1.28)	0.713
**Missing**	1485 (11.5)	69 (9.8)				
**Diabetes during pregnancy (%) [n=219/12773]**	209 (1.6)	10 (1.4)	0.86 (0.45-1.63)	0.182	0.87 (0.46-1.67)	0.423
**Missing**	759 (5.9)	30 (4.3)				
**Age at first live birth (%) [n=13304]**				0.014		0.009
**≤20**	2083 (16.2)	91 (13.0)	Ref		Ref	
**21-25**	6042 (47.0)	316 (45.0)	1.20 (0.94-1.52)		1.22 (0.95-1.56)	
**26-30**	3278 (25.5)	217 (30.9)	**1.52 (1.18-1.95)**		**1.56 (1.20-2.04)**	
**≥31**	1213 (9.4)	64 (9.1)	1.21 (0.87-1.68)		1.28 (0.90-1.81)	
**Missing**	244 (1.9)	14 (2.0)				
**Number of children (%) [n=13562]**				0.742		0.585
**0**	190 (1.5)	13 (1.9)	Ref		Ref	
**1**	2209 (17.2)	113 (16.1)	0.75 (0.41-1.35)		0.28 (0.04-2.24)	
**2**	6023 (46.8)	336 (47.9)	0.82 (0.46-1.45)		0.30 (0.04-2.35)	
**≥3**	4438 (34.5)	240 (34.2)	0.79 (0.44-1.41)		0.31 (0.04-2.46)	
**Number of stillbirths (%) [n=13562]**				0.963		0.948

**None**	12461 (96.9)	680 (96.9)	Ref		Ref	
**Any**	399 (3.1)	22 (3.1)	1.01 (0.65-1.56)		1.02 (0.65-1.58)	
**Number of miscarriages or abortions [n=13562]**						
**0**	9646 (75.0)	510 (72.6)	Ref	0.468	Ref	0.781
**1**	2412 (18.8)	144 (20.5.)	1.13 (0.93-1.37)		1.07 (0.88-1.31)	
**2**	574 (4.5)	32 (4.6)	1.05 (0.73-1.52)		1.00 (0.69-1.45)	
**≥3**	228 (1.8)	16 (2.3)	1.33 (0.79-2.22)		1.25 (0.74-2.10)	

*Adjusted for age, education level, occupational social class, smoking status, alcohol consumption, physical activity, BMI, family history of cancer, prevalent MI, prevalent stroke, prevalent diabetes, diabetes during pregnancy, age at first live birth, number of children, number of still births and number of miscarriages or abortion.

Table S2. Baseline characteristics of 13,562 women of the EPIC-Norfolk according to incident colorectal cancer status and corresponding unadjusted and adjusted odds ratios with 95% CIs

	Incident colorectal cancer		Unadjusted OR (95% CI)	P-value	Adjusted OR* (95% CI)	P-value
	No [n=13096]	Yes [n=466]				
**Median age years (IQR) [n=13562]**	**57.9 (16.1)**	**63.5 (13.8)**	**1.05 (1.04-1.06)**	<0.001	**1.05 (1.03-1.06)**	<0.001
**Education (%) [n=13562]**						
**No qualification**	5854 (44.7)	243 (52.1)	Ref	0.007	Ref	0.671
**0 Level**	1543 (11.8)	47 (10.1)	0.73 (0.53-1.01)		0.95 (0.68-1.31)	
**A Level**	4450 (34.0)	146 (31.3)	**0.79 (0.64-0.97)**		0.97 (0.77-1.22)	
**Degree**	1249 (9.5)	30 (6.4)	**0.58 (0.39-0.85)**		0.77 (0.50-1.17)	
**Occupational Social Class (%) [n=13190]**						
**Professional**	795 (6.1)	25 (5.4)	Ref	0.905	Ref	0.686
**Manager**	4238 (32.4)	147 (31.5)	1.10 (0.72-1.70)		1.04 (0.67-1.61)	
**Skilled Non-manual**	2441 (18.6)	84 (18.0)	1.09 (0.70-1.72)		0.88 (0.55-1.40)	
**Skilled Manual**	2844 (21.7)	110 (23.6)	1.23 (0.79-1.91)		1.11 (0.70-1.76)	
**Semi-Skilled**	1843 (14.1)	62 (13.3)	1.07 (0.67-1.72)		0.88 (0.54-1.44)	
**Non-Skilled**	578 (4.4)	23 (4.9)	1.27 (0.71-2.25)		0.96 (0.53-1.75)	
**Missing**	357 (2.7)	15 (3.2)				
**Smoking History (%) [n=13441]**						
**Never**	7228 (55.2)	250 (53.6)	Ref	0.834	Ref	0.461
**Ex-smoker**	16981 (31.8)	155 (33.3)	1.06 (0.79-1.42)		1.03 (0.83-1.27)	

**Currently smoking**	1581 (12.1.)	58 (12.4)	1.08 (0.88-1.32)		1.22 (0.91-1.65)	
**Missing**	118 (0.9)	3 (0.6)				
**Alcohol (g) (%) [n=11035]**						
**0**	2597 (19.8)	114 (24.5)	Ref		Ref	
**0.1-4.9**	4180 (31.9)	142 (30.5)	0.77 (0.60-1.00)		0.87 (0.67-1.12)	
**5-14.9**	2987 (22.8)	87 (18.7)	**0.66 (0.50-0.88)**	0.107	0.79 (0.59-1.07)	0.707
**15-29.9**	656 (5.0)	25 (5.4)	0.87 (0.56-1.35)		1.02 (0.65-1.61)	
**≥30**	240 (1.8)	7 (1.5)	0.66 (0.31-1.44)		0.84 (0.38-1.86)	
**Missing**	2436 (18.6)	91 (19.5)				
**Physical activity (%) [n=13562]**						
**Active**	4921 (37.6)	134 (28.8)	Ref	<0.001	Ref	0.180
**Inactive**	8175 (62.4)	332 (71.2)	**1.49 (1.21-1.83)**		1.16 (0.94-1.43)	
**BMI (kg/m²) (%) [n=11414]**						
**Normal**	4818 (36.8)	131 (28.1)	Ref		Ref	
**Underweight**	70 (0.5)	2 (0.4)	1.05 (0.26-4.33)		1.04 (0.25-4.32)	
**Overweight**	4276 (32.7)	179 (38.4)	**1.54 (1.22-1.94)**	<0.001	**1.34 (1.06-1.69)**	0.005
**Obese**	1421 (10.9)	48 (10.3)	1.24 (0.89-1.74)		1.07 (0.76-1.51)	
**Morbidly obese**	440 (3.4)	29 (6.2)	**2.42 (1.60-3.67)**		**2.27 (1.48-3.47)**	
**Missing**	2071 (15.8)	77 (16.5)				
**Family history of cancer (%) [n=5295/13561]**	5091 (38.9)	204 (43.8)	**1.22 (1.02-1.48)**	0.104	**1.20 (0.99-1.45)**	0.167
**Missing**						

	1 (0.0)	0 (0.0)				
**Prevalent MI (%) [n=200/13557]**	193 (1.5)	7 (1.5)	1.02 (0.48-2.18)	0.956	0.75 (0.35-1.61)	0.156
**Missing**	4 (0.0)	1 (0.2)				
**Prevalent stroke (%) [n=144/13559]**	143 (1.1)	1 (0.2)	0.20 (0.03-1.40)	0.266	0.13 (0.02-0.97)	0.137
**Missing**	3 (0.0)	0 (0.0)				
**Prevalent Diabetes (%) [n=226/13557]**	218 (1.7)	8 (1.7)	1.03 (0.51-2.10)	0.996	0.75 (0.37-1.55)	0.745
**Missing**	5 (0.0)	0 (0.0)				
**Hypertension during pregnancy (%) [n =2919/12008]**	2807 (21.4)	112 (24.0)	1.17 (0.93-1.45)	0.399	1.15 (0.92-1.45)	0.154
**Missing**	1501 (11.5)	53 (11.4)				
**Diabetes during pregnancy (%) [n=219/12773]**	210 (1.6)	9 (1.9)	1.24 (0.63-2.44)	0.049	1.40 (0.71-2.78)	0.238
**Missing**	750 (5.7)	39 (8.4)				
**Age at first live birth (%) [n=13304]**				0.547		0.686
**≤20**	2104 (16.1)	70 (15.0)	Ref		Ref	
**21-25**	6152 (47.0)	206 (44.2)	1.01 (0.76-1.33)		0.99(0.74-1.31)	
**26-30**	3366 (25.7)	129 (27.7)	1.15 (0.86-1.55)		1.13 (0.83-1.56)	
**≥31**	1226 (9.4)	51 (10.9)	1.25 (0.87-1.81)		1.22 (0.83-1.82)	
**Missing**	248 (1.9)	10 (2.1)				
**Number of children (%) [n=13562]**				0.125		0.756
**0**	195 (1.5)	8 (1.7)	Ref		Ref	
**1**	2230 (17.0)	92 (19.7)	1.01 (0.48-2.10)		0.78 (0.16-3.90)	
**2**	6165 (47.1)	194 (41.6)	0.77 (0.37-1.58)		0.73 (0.15-3.63)	

**≥3**	4506 (34.4)	172 (36.9)	0.93 (0.45-1.92)		0.82 (0.16-4.06)	
**Number of stillbirths (%) [n=13562]**						
**None**	12691 (96.9)	450 (96.6)	Ref	0.677	Ref	0.869
**Any**	405 (3.1)	16 (3.4)	1.11 (0.67-1.85)		0.96 (0.57-1.60)	
**Number of miscarriages or abortions [n=13562]**						
**0**	9799 (74.8)	357 (76.6)	Ref		Ref	
**1**	2472 (18.9)	84 (18.0)	0.93 (0.73-1.19)	0.747	1.00 (0.78-1.28)	0.938
**2**	587 (4.5)	19 (4.1)	0.88 (0.56-1.42)		0.96 (0.59-1.54)	
**≥3**	238 (1.8)	6 (1.3)	0.69 (0.31-1.57)		0.77 (0.34-1.76)	

*Adjusted for age, education level, Townsend Deprivation Index, smoking status, alcohol consumption, physical activity, BMI, family history of cancer, prevalent MI, prevalent stroke, prevalent diabetes, diabetes during pregnancy, age at first live birth, number of children, number of still births and number of miscarriages or abortion.

Table S3. Baseline characteristics of 13,562 women of the EPIC-Norfolk according to incident lung cancer status and corresponding unadjusted and adjusted odds ratios with 95% CIs

	Incident lung cancer		Unadjusted OR (95% CI)	P-value	Adjusted OR* (95% CI)	P-value
	No [n=13315]	Yes [n=247]				
**Median age years (IQR) [n=13562]**	**58.0 (16.2)**	**62.9 (14.2)**	**1.04 (1.03-1.05)**	<0.001	**1.04 (1.03-1.06)**	<0.001
**Education (%) [n=13562]**						0.064
**No qualification**	5949 (44.7)	148 (59.9)	Ref	<0.001	Ref	
**0 Level**	1563 (11.7)	27 (10.9)	0.69 (0.46-1.05)		0.92 (0.60-1.43)	
**A Level**	4530 (34.0)	66 (26.7)	**0.59 (0.44-0.79)**		0.84 (0.60-1.16)	
**Degree**	1273 (9.6)	6 (2.4)	**0.19 (0.08-0.43)**		0.32 (0.13-0.75)	
**Occupational Social Class (%) [n=13190]**						0.213
**Professional**	808 (6.1)	12 (4.9)	Ref	0.019	Ref	
**Manager**	4317 (32.4)	68 (27.5)	1.06 (0.57-1.97)		0.81 (0.43-1.52)	
**Skilled Non-manual**	2491 (18.7)	34 (13.8)	0.92 (0.47-1.78)		0.53 (0.27-1.06)	
**Skilled Manual**	2892 (21.7)	62 (25.1)	1.44 (0.77-2.69)		0.88 (0.46-1.69)	
**Semi-Skilled**	1861 (14.0)	44 (17.8)	1.59 (0.84-3.03)		0.88 (0.45-1.74)	
**Non-Skilled**	582 (4.4)	19 (7.7)	**2.20 (1.06-4.56)**		1.11 (0.51-2.42)	
**Missing**	364 (2.7)	8 (3.2)				
**Smoking History (%) [n=13441]**						<0.001
**Never**	7431 (55.8)	47 (19.0)	Ref	<0.001	Ref	
**Ex-smoker**	4241 (31.9)	116 (47.0)	**3.09 (2.16-4.43)**		**2.91 (2.02-4.20)**	

**Currently smoking**	1523 (11.4)	83 (33.6)	**12.04 (8.54-16.98)**		**13.22 (9.26-18.89.)**	
**Missing**	120 (0.9)	1 (0.4)				
**Alcohol (g) (%) [n=11035]**						0.654
**0**	2664 (20.0)	47 (19.0)	Ref		Ref	
**0.1-4.9**	4249 (31.9)	73 (29.6)	0.97 (0.67-1.41)		1.15 (0.78-1.68)	
**5-14.9**	3027 (22.7)	47 (19.0)	0.88 (0.59-1.32)	0.049	1.05 (0.68-1.62)	
**15-29.9**	668 (5.0)	13 (5.3)	1.10 (0.59-2.05)		1.04 (0.54-1.98)	
**≥30**	245 (1.8)	2 (0.8)	0.46 (0.11-1.92)		0.50 (0.12-2.13)	
**Missing**	2462 (18.5)	65 (26.3)				
**Physical activity (%) [n=13562]**						0.006
**Active**	4997 (37.5)	58 (23.5)	Ref	<0.001	Ref	
**Inactive**	8318 (62.5)	189 (76.5)	**1.96 (1.46-2.63)**		**1.55 (1.13-2.12)**	
**BMI (kg/m²) (%) [n=11414]**						0.204
**Normal or Underweight**	4948 (37.2)	73 (29.6)	Ref		Ref	
**Pre-obesity**	4365 (32.8)	90 (36.4)	**1.40 (1.02-1.91)**		1.36 (0.99-1.88)	
**Obesity Class I**	1442 (10.8)	27 (10.9)	**1.27 (0.81-1.98)**	0.034	1.17 (0.74-1.85)	
**Obesity Class II +**	464 (3.5)	5 (2.0)	0.73 (0.29-1.82)		0.69 (0.27-1.74)	
**Missing**	2096 (15.7)	52 (21.1)				
**Family history of cancer (%) [n=5295/13561]**	5192 (39.0)	103 (41.7)	1.12 (0.87-1.44)	0.689	1.09 (0.84-1.42)	0.811
**Missing**	1 (0.0)	0 (0.0)				

**Prevalent MI (%) [n=200/13557]**	195 (1.5)	5 (2.0)	1.39 (0.57-3.41)	0.772	1.01 (0.40-2.55)	1.00
**Missing**	5 (0.0)	0 (0.0)				
**Prevalent stroke (%) [n=144/13559]**	142 (1.1)	2 (0.9)	0.76 (0.19-3.07)	0.927	0.45 (0.11-1.89)	0.556
**Missing**	3 (0.0)	0 (0.0)				
**Prevalent Diabetes (%) [n=226/13557]**	220 (1.7)	6 (2.4)	1.48 (0.65-3.37)	0.644	1.32 (0.56-3.11)	0.820
**Missing**	5 (0.0)	0 (0.0)				
**Hypertension during pregnancy (%) [n =2919/12008]**	2875 (21.6)	44 (17.8)	0.84 (0.60-1.18)	0.025	0.96 (0.68-1.35)	0.122
**Missing**	1513 (11.4)	41 (16.6)				
**Diabetes during pregnancy (%) [n=219/12773]**	215 (1.6)	4 (1.6)	1.01 (0.37-2.73)	0.985	1.20 (0.43-3.35)	0.421
**Missing**	774 (5.8)	15 (6.1)				
**Age at first live birth (%) [n=13304]**						0.494
**≤20**	2114 (15.9)	60 (24.3)	Ref		Ref	
**21-25**	6255 (47.0)	103 (41.7)	**0.58 (0.42-0.80)**	0.008	0.78 (0.56-1.10)	
**26-30**	3441 (25.8)	54 (21.9)	**0.55 (0.38-0.80)**		0.90 (0.60-1.36)	
**≥31**	1253 (9.4)	24 (9.7)	0.68 (0.42-1.09)		1.08 (0.63-1.84)	
**Missing**	252 (1.9)	6 (2.4)				
**Number of children (%) [n=13562]**						0.500
**0**	199 (1.5)	4 (1.6)	Ref		Ref	
**1**	2275 (17.1)	47 (19.0)	1.03 (0.37-2.88)	0.045	1.22 (0.20-7.39)	
**2**	6265 (47.1)	94 (38.1)	0.75 (0.27-2.05)		1.20 (0.20-7.31)	
**≥3**	4576 (34.4)	102 (41.3)	1.11 (0.40-3.04)		1.49 (0.25-9.06)	
**Number of stillbirths (%) [n=13562]**				0.538		0.336

**None**	12900 (96.9)	241 (97.6)	Ref		Ref	
**Any**	415 (3.1)	6 (2.4)	0.77 (0.34-1.75)		0.67 (0.29-1.53)	
**Number of miscarriages or abortions [n=13562]**						0.570
**0**	9969 (74.9)	187 (75.7)	Ref	0.482	Ref	
**1**	2516 (18.9)	40 (16.2)	0.85 (0.60-1.20)		0.87 (0.61-1.25)	
**2**	591 (4.4)	15 (6.1)	1.35 (0.79-2.30)		1.36 (0.78-2.38)	
**≥3**	239 (1.8)	5 (2.0)	1.12 (0.46-2.74)		1.08 (0.42-2.74)	

*Adjusted for age, education level, occupational social class, smoking status, alcohol consumption, physical activity, BMI, family history of cancer, prevalent MI, prevalent stroke, prevalent diabetes, diabetes during pregnancy, age at first live birth, number of children, number of still births and number of miscarriages or abortion.

Table S4. Baseline characteristics of 13,562 women of the EPIC-Norfolk according to incident ovarian cancer status and corresponding unadjusted and adjusted odds ratios with 95% CIs

	Incident ovarian cancer		Unadjusted OR (95% CI)	P-value	Adjusted OR* (95% CI)	P-value
	No [n=13374]	Yes [n=188]				
**Median (IQR) age years [n=13562]**	**58.0 (16.2)**	**59.5 (12.7)**	**1.01 (1.00-1.03)**	0.184	1.00 (0.99-1.02)	0.650
**Education (%) [n=13562]**						
**No qualification**	5999 (44.9)	98 (52.1)	Ref	0.247	Ref	0.385
**0 Level**	1572 (11.8)	18 (9.6)	0.70 (0.42-1.16)		0.72 (0.43-1.21)	
**A Level**	4541 (34.0)	55 (29.3)	**0.74 (0.53-1.03)**		0.75 (0.52-1.08)	
**Degree**	1262 (9.4)	17 (9.0)	0.83 (0.49-1.39)		0.83 (0.46-1.48)	
**Occupational Social Class (%) [n=13190]**						
**Professional**	812 (6.1)	8 (4.3)		0.953		0.950
**Manager**	4325 (32.3)	60 (31.9)	Ref		Ref	
**Skilled Non-manual**	2488 (18.6)	37 (19.7)	1.41 (0.67-2.96)		1.38 (0.65-2.91)	
**Skilled Manual**	2911 (21.8)	43 (22.9)	1.51 (0.70-3.26)		1.42 (0.64-3.11)	
**Semi-Skilled**	1878 (14.0)	27 (14.4)	1.50 (0.70-3.20)		1.34 (0.61-2.95)	
**Non-Skilled**	594 (4.4)	7 (3.7)	1.46 (0.66-3.23)		1.25 (0.55-2.86)	
**Missing**	366 (2.7)	6 (3.2)	1.20 (0.43-3.32)		0.96 (0.34-2.76)	
**Smoking History (%) [n=13441]**						
**Never**	7375 (55.1)	103 (54.8)	Ref	0.946	Ref	0.910
**Ex-smoker**	4262 (31.9)	62 (33.0)	1.04 (0.76-1.43)		1.07 (0.67-1.72)	
**Currently smoking**	1617 (12.1)	22 (11.7)	0.97 (0.61-1.55)		1.05 (0.76-1.45)	

**Missing**	120 (0.9)	1 (0.5)				
**Alcohol (g) (%) [n=11035]**						
**0**	2661 (19.9)	50 (26.6)	Ref		Ref	
**0.1-4.9**	4272 (31.9)	50 (26.6)	0.62 (0.42-0.93)		0.64 (0.43-0.95)	
**5-14.9**	3034 (22.7)	40 (21.3)	0.70 (0.46-1.07)	0.240	0.75 (0.48-1.16)	0.347
**15-29.9**	674 (5.0)	7 (3.7)	0.55 (0.25-1.23)		0.59 (0.26-1.34)	
**≥30**	243 (1.8)	4 (2.1)	0.88 (0.31-2.45)		0.96 (0.34-2.74)	
**Missing**	2490 (18.6)	37 (19.7)				
**Physical activity (%) [n=13562]**						
**Active**	8393 (62.8)	114 (60.6)	Ref	0.551	Ref	0.224
**Inactive**	4981 (37.2)	74 (39.4)	0.91 (0.68-1.23)		0.83 (0.61-1.13)	
**BMI (kg/m²) (%) [n=11414]**						
**Normal or Underweight**	4965 (37.1)	56 (29.8)	Ref		Ref	
**Pre-obesity**	4385 (32.8)	70 (37.2)	1.42 (0.99-2.02)		1.37 (0.96-1.97)	
**Obesity Class I**	1445 (10.8)	24 (12.8)	1.47 (0.91-2.38)	0.340	1.41 (0.86-2.30)	0.473
**Obesity Class II +**	462 (3.5)	7 (3.7)	1.34 (0.61-2.96)		1.30 (0.58-2.91)	
**Missing**	2117 (15.8)	31 (16.5)				
**Family history of cancer (%) [n=5295/13561]**	5206 (38.9)	89 (47.3)	**1.41 (1.06-1.88)**	0.065	**1.42 (1.06-1.89)**	0.063
**Missing**	1 (0.0)	0 (0.0)				
**Prevalent MI (%) [n=200/13557]**	197 (1.5)	3 (1.6)	1.08 (0.34-3.42)	0.991	1.01 (0.32-3.24)	1.00
**Missing**	5 (0.0)	0 (0.0)				

**Prevalent stroke (%) [n=144/13559]**	144 (1.1)	0 (0.0)	----	----	----	----
**Missing**	3 (0.0)	0 (0.0)				
**Prevalent Diabetes (%) [n=226/13557]**	223 (1.7)	3 (1.6)	0.96 (0.30-3.01)	0.997	0.85 (0.27-2.75)	0.966
**Missing**	5 (0.0)	0 (0.0)				
**Hypertension during pregnancy (%) [n =2919/12008]**	2868 (21.4)	51 (27.1)	1.36 (0.98-1.90)	0.172	1.30 (0.93-1.83)	0.183
**Missing**	1534 (11.5)	20 (10.6)				
**Diabetes during pregnancy (%) [n=219/12773]**	215 (1.6)	4 (2.1)	1.37 (0.50-3.73)	0.239	1.39 (0.50-3.82)	0.266
**Missing**	773 (5.8)	16 (8.5)				
**Age at first live birth (%) [n=13304]**						
**≤20**	2150 (16.1)	24 (12.8)	Ref	0.038	Ref	0.074
**21-25**	6271 (46.9)	87 (46.3)	1.24 (0.79-1.96)		1.28 (0.80-2.04)	
**26-30**	3451 (25.8)	44 (23.4)	1.14 (0.69-1.88)		1.20 (0.71-2.02)	
**≥31**	1247 (9.3)	30 (16.0)	**2.16 (1.25-3.70)**		**2.19 (1.21-3.95)**	
**Missing**	255 (1.9)	3 (1.6)				
**Number of children (%) [n=13562]**						
**0**	201 (1.5)	2 (1.1)	Ref	0.392	Ref	0.824
**1**	2283 (17.1)	39 (20.7)	1.72 (0.41-7.16)		1.60 (0.14-18.76)	
**2**	6268 (46.9)	91 (48.4)	1.46 (0.36-5.97)		1.57 (0.13-18.42)	
**≥3**	4622 (34.6)	56 (29.8)	1.22 (0.30-5.03)		1.36 (0.12-15.88)	

**Number of stillbirths (%) [n=13562]**						
**None**	12955 (96.9)	186 (98.9)	Ref	0.122	Ref	0.111
**Any**	419 (3.1)	2 (1.1)	0.33 (0.08-1.34)		0.32 (0.08-1.30)	
**Number of miscarriages or abortions [n=13562]**						
**0**	10008 (74.8)	148 (78.7)	Ref	0.645	Ref	0.789
**1**	2525 (18.9)	31 (16.5)	0.83 (0.56-1.23)		0.86 (0.58-1.28)	
**2**	600 (4.5)	6 (3.2)	0.68 (0.30-1.54)		0.72 (0.31-1.65)	
**≥3**	241 (1.8)	3 (1.6)	0.84 (0.27-2.66)		0.88 (0.28-2.82)	

*Adjusted for age, education level, occupational social class, smoking status, alcohol consumption, physical activity, BMI, family history of cancer, prevalent MI, prevalent diabetes, diabetes during pregnancy, age at first live birth, number of children, number of still births and number of miscarriages or abortion.

Table S5. Baseline characteristics of 13,562 women of the EPIC-Norfolk according to incident endometrial cancer status and corresponding unadjusted and adjusted odds ratios with 95% CIs

	**Incident endometrial cancer**		**Unadjusted OR (95% CI)**	**P-value**	**Adjusted OR* (95% CI)**	**P-value**
	**No [n=13399]**	**Yes [n=163]**				
**Median (IQR) age years [n=13562]**	**58.1 (16.2)**	**56.7 (14.00)**	**1.00 (0.98-1.01)**	0.610	0.99 (0.97-1.01)	0.256
**Education (%) [n=13562]**						0.327
**No qualification**	6019 (44.9)	78 (47.9)	Ref		Ref	
**0 Level**	1565 (11.7)	25 (15.3)	1.23 (0.78-1.94)	0.258	1.26 (0.78-2.03)	
**A Level**	4551 (34.0)	45 (27.6)	**0.76 (0.53-1.10)**		0.79 (0.53-1.18)	
**Degree**	1264 (9.4)	15 (9.2)	0.92 (0.53-1.60)		0.99 (0.53-1.84)	
**Occupational Social Class (%) [n=13190]**						0.190
**Professional**	816 (6.1)	4 (2.5)	Ref		Ref	
**Manager**	4336 (32.4)	49 (30.1)	2.31 (0.83-6.41)		2.36 (0.84-6.60)	
**Skilled Non-manual**	2494 (18.6)	31 (19.0)	2.54 (0.89-7.21)	0.108	2.58 (0.89-7.46)	
**Skilled Manual**	2913 (21.7)	41 (25.2)	**2.87 (1.03-8.04)**		2.70 (0.94-7.74)	
**Semi-Skilled**	1874 (14.0)	31 (19.0)	**3.38 (1.19-9.59)**		**3.06 (1.05-8.95)**	
**Non-Skilled**	599 (4.5)	2 (1.2)	0.68 (0.12-3.73)		0.60 (0.11-3.38)	
**Missing**	367 (2.7)	5 (3.1)				
**Smoking History (%) [n=13441]**						<0.001
**Never**	7361 (54.9)	117 (71.8)	Ref		Ref	
**Ex-smoker**	4286 (32.0)	38 (23.3)	**0.56 (0.39-0.81)**	<0.001	**0.28 (0.13-0.61)**	
**Currently smoking**	1632 (12.2)	7 (4.3)	**0.27 (0.13-0.58)**		**0.53 (0.37-0.78)**	

Missing	120 (0.9)	1 (0.6)				
Alcohol (g) (%) [n=11035]						0.873
0	2673 (19.9)	38 (23.3)	Ref		Ref	
0.1-4.9	4265 (31.8)	57 (35.0)	0.94 (0.62-1.42)		1.01 (0.66-1.54)	
5-14.9	3045 (22.7)	29 (17.8)	0.67 (0.41-1.09)	0.534	0.89 (0.53-1.47)	
15-29.9	675 (5.0)	6 (3.7)	0.63 (0.26-1.49)		0.98 (0.40-2.37)	
≥30	243 (1.8)	4 (2.5)	1.16 (0.41-3.27)		1.78 (0.61-5.16)	
Missing	2498 (18.6)	29 (17.8)				
Physical activity (%) [n=13562]						0.556
Active	4994 (37.3)	61 (37.4)	Ref	0.968	Ref	
Inactive	4405 (62.7)	102 (62.6)	0.99 (0.72-1.37)		0.90 (0.65-1.27)	
BMI (kg/m²) (%) [n=11414]						<0.001
Normal or Underweight	4982 (37.2)	39 (23.9)	Ref		Ref	
Pre-obesity	4411 (32.9)	44 (27.0)	1.27 (0.83-1.97)		1.26 (0.81-1.95)	
Obesity Class I	1435 (10.7)	34 (20.9)	**3.03 (1.90-4.81)**	<0.001	**3.00 (1.86-4.82)**	
Obesity Class II +	448 (3.3)	21 (12.9)	**5.99 (3.49-10.27)**		**5.90 (3.36-10.37)**	
Missing	2123 (15.8)	25 (15.3)				
Family history of cancer (%) [n=5295/13561]	5229 (39.0)	66 (40.5)	1.06 (0.78-1.46)	0.930	1.09 (0.79-1.50)	0.872
Missing	1 (0.0)	0 (0.0)				
Prevalent MI (%) [n=200/13557]	196 (1.5)	4 (2.5)	1.69 (0.62-4.62)	0.588	1.61 (0.57-4.56)	0.672
Missing	5 (0.0)	0 (0.0)				

**Prevalent stroke (%) [n=144/13559]**	141 (1.1)	3 (1.8)	1.76 (0.56-5.59)	0.629	1.77 (0.54-5.83)	0.646
**Missing**	3 (0.0)	0 (0.0)				
**Prevalent Diabetes (%) [n=226/13557]**	220 (1.6)	6 (3.7)	2.29 (1.00-5.23)	0.145	1.70 (0.72-4.05)	0.484
**Missing**	5 (0.0)	0 (0.0)				
**Hypertension during pregnancy (%) [n =2919/12008]**	2875 (21.5)	44 (27.0)	1.42 (0.99-2.03)	0.120	1.16 (0.80-1.67)	0.577
**Missing**	1532 (11.4)	22 (13.5)				
**Diabetes during pregnancy (%) [n=219/12773]**	214 (1.6)	5 (3.1)	1.95 (0.79-4.79)	0.346	1.52 (0.60-3.85)	0.598
**Missing**	780 (5.8)	9 (5.5)				
**Age at first live birth (%) [n=13304]**						0.578
**≤20**	2152 (16.1)	22 (13.5)	Ref	0.733	Ref	
**21-25**	6282 (46.9)	76 (46.6)	1.18 (0.73-1.91)		1.22 (0.75-1.98)	
**26-30**	3451 (25.8)	44 (27.0)	1.25 (0.75-2.09)		1.36 (0.80-2.32)	
**≥31**	1258 (9.4)	19 (11.7)	1.48 (0.80-2.74)		1.66 (0.88-3.15)	
**Missing**	256 (1.9)	2 (1.2)				
**Number of children (%) [n=13562]**						
**0**	203 (1.5)	0 (0.0)				
**1**	2294 (17.1)	28 (17.2)	--------		--------	
**2**	6277 (46.8)	82 (50.3)				
**3 or more**	4625 (34.5)	53 (32.5)				
**Number of stillbirths (%) [n=13562]**				0.978		0.971
**None**	12983 (96.9)	158 (96.9)	Ref		Ref	

**Any**	416 (3.1)	5 (3.1)	0.99 (0.40-2.42)		1.02 (0.41-2.52)	
**Number of miscarriages or abortions [n=13562]**						0.496
**0**	10029 (74.8)	127 (77.9)	Ref	0.459	Ref	
**1**	2526 (18.9)	30 (18.4)	0.94 (0.63-1.40)		0.97 (0.64-1.46)	
**2**	603 (4.5)	3 (1.8)	0.39 (0.13-1.24)		0.40 (0.13-1.28)	
**≥3**	241 (1.8)	3 (1.8)	0.98 (0.31-3.11)		0.92 (0.28-2.95)	

*Adjusted for age, education level, occupational social class, smoking status, alcohol consumption, physical activity, BMI, family history of cancer, prevalent MI, prevalent stroke prevalent diabetes, diabetes during pregnancy, age at first live birth, number of still births and number of miscarriages or abortion.

Table S6. Unadjusted and adjusted odds ratios and corresponding 95% CIs for HDP and incident cancer and site-specific cancers in the subgroup of participants with high BMI

	**Hypertension during pregnancy [n=1573]**			
	**Unadjusted OR (95% CI)**	**P-value**	**Adjusted OR[1] (95% CI)**	**P-value**
**Incident Cancer [n=1330]**	1.12 (0.98-1.30)	0.251	**1.17 (1.01-1.35)**	0.099
**Breast [n=369]**	1.07 (0.84-1.37)	0.859	1.05 (0.82-1.35)	0.687
**Colorectal [n=256]**	1.28 (0.96-1.70)	0.206	**1.37 (1.02-1.83)**[2]	0. 113
**Lung [n=122]**	0.89 (0.57-1.38)	0.752	1.00 (0.63-1.59)	0.982
**Ovarian [n=101]**	1.47 (0.95-2.26)	0.159	1.50 (0.97-2.34)[3]	0.114
**Endometrial [n=99]**	1.38 (0.88-2.16)	0.364	1.30 (0.83-2.06)[4]	0.513

[1]Adjusted for age, education level, occupational social class, smoking status, alcohol consumption, physical activity, family history of cancer, prevalent MI, prevalent stroke, prevalent diabetes, diabetes during pregnancy, age at first live birth, number of children, number of still births and number of miscarriages or abortion.

[2] Adjusted for age, education level, occupational social class, smoking status, alcohol consumption, physical activity, family history of cancer, prevalent MI, prevalent stroke, prevalent diabetes, diabetes during pregnancy, number of children, number of still births and number of miscarriages or abortion.

[3]Adjusted for age, education level, occupational social class, smoking status, alcohol consumption, physical activity, family history of cancer, prevalent MI, prevalent diabetes, diabetes during pregnancy, age at first live birth, number of children, number of still births and number of miscarriages or abortion.

[4]Adjusted for age, education level, occupational social class, smoking status, alcohol consumption, physical activity, family history of cancer, prevalent MI, prevalent stroke, prevalent diabetes, diabetes during pregnancy, age at first live birth, number of still births and number of miscarriages or abortion.
